# Preparation of Catechin Nanoemulsion from Oolong Tea Leaf Waste and Its Inhibition of Prostate Cancer Cells DU-145 and Tumors in Mice

**DOI:** 10.3390/molecules26113260

**Published:** 2021-05-28

**Authors:** Yu-Hsiang Lin, Chi-Chung Wang, Ying-Hung Lin, Bing-Huei Chen

**Affiliations:** 1 Department of Food Science, Fu Jen Catholic University, New Taipei City 242, Taiwan; sonyboys67@gmail.com; 2 Graduate Institute of Biomedical and Pharmaceutical Science, Fu Jen Catholic University, New Taipei City 242, Taiwan; 075006@mail.fju.edu.tw (C.-C.W.); 084952@mail.fju.edu.tw (Y.-H.L.); 3 Department of Nutrition, China Medical University, Taichung 404, Taiwan

**Keywords:** catechin nanoemulsion, paclitaxel, Oolong tea leaf waste, prostate cancer cell, mice tumor

## Abstract

Anti-cancer activity of catechin nanoemulsions prepared from Oolong tea leaf waste was studied on prostate cancer cells DU-145 and DU-145-induced tumors in mice. Catechin nanoemulsions composed of lecithin, Tween-80 and water in an appropriate proportion was prepared with high stability, particle size of 11.3 nm, zeta potential of −67.2 mV and encapsulation efficiency of 83.4%. Catechin nanoemulsions were more effective than extracts in inhibiting DU-145 cell growth, with the IC_50_ being 13.52 and 214.6 μg/mL, respectively, after 48 h incubation. Furthermore, both catechin nanoemulsions and extracts could raise caspase-8, caspase-9 and caspase-3 activities for DU-145 cell apoptosis, arresting the cell cycle at S and G2/M phases. Compared to control, catechin nanoemulsion at 20 μg/mL and paclitaxel at 10 μg/mL were the most effective in reducing tumor volume by 41.3% and 52.5% and tumor weight by 77.5% and 90.6% in mice, respectively, through a decrease in EGF and VEGF levels in serum.

## 1. Introduction

Oolong tea, a semi-fermented tea widely consumed in Asian countries, has been shown to contain an abundant amount of catechins, including epigallocatechin gallate (EGCG), epicatechin gallate (ECG), epigallocatechin (EGC), epicatechin (EC), gallocatechin gallate (GCG) and catechin (C) [1]. Among the various catechins in Oolong tea, EGCG was reported to be present in the largest amount while GCG was the lowest amount [2]. Most importantly, catechins have been demonstrated to be protective against many types of chronic diseases such as inflammation, atherosclerosis and prostate cancer [3].

Oolong tea beverage represents a vital product in the Taiwan beverage industry due to an increasing demand by consumers annually. Interestingly, Oolong tea leaf waste, a by-product produced during the processing of Oolong tea beverage, was also shown to contain a significant amount of catechins [4]. Due to the great contribution of catechins to human health, it would be a great advantage to the food or drug industry if catechins can be further extracted from Oolong tea leaf waste and processed into functional food or even botanic drugs for possible clinical application in the treatment of chronic diseases. However, the high instability and poor bioavailability of catechins in vivo have limited their potential for further development into a botanic drug [5]. Nevertheless, through preparation of nanoemulsions or microemulsions for encapsulation of catechins, both the catechin stability and bioavailability in vivo can be greatly enhanced to improve the anti-cancer effect substantially [6].

Accordingly, both nanoemulsions and microemulsions are composed of oil, water, surfactant or co-surfactant in an appropriate proportion, with the former size ranging from 10 to 100 nm and the latter size from 2 to 100 nm [7]. Furthermore, microemulsion is thermodynamically more stable than nanoemulsion, with a higher surface-to-mass ratio [7]. The application of drug- or natural bioactive compound-loaded nanoemulsion or microemulsion in inhibiting cancer cell growth has been extensively studied. For example, Huang et al. [8] prepared a lycopene–nanogold nanoemulsion with the average particle size being 21.3 nm by TEM analysis and demonstrated that it was effective in inhibiting the growth of colon cancer cell HT-29 through passive targeting by enhanced permeability and retention (EPR) effect. In other words, nanoemulsion is able to diffuse from the extracellular matrix into the cytoplasm and nucleus for antitumor efficiency [8].

In the literature reports, most studies deal with the effects of catechin standards or green tea extracts on the growth of cancer cells. For instance, Carvalho et al. [9] reported that the green tea extract was effective in inhibiting growth of kidney cancer cells A-497 and 769-P, with half the maximal inhibitory concentration (IC_50_) being 54 μg/mL and 129 μg/mL, respectively. In a later study, Singh et al. [10] further illustrated that EGCG, the most abundant catechin in green tea extract, was effective in inhibiting the growth of cervical cancer cells SiHa through an increase in both caspase-9 and caspase-3 activities. In a previous study, Tsai and Chen [6] prepared a highly stable catechin nanoemulsion from green tea leaf waste and reported that it was effective in inhibiting the growth of prostate cancer cells PC-3 with the IC_50_ being 8.5 μg/mL through activation of caspase-8, caspase-9 and caspase-3. However, the inhibition effect of catechin nanoemulsion prepared from Oolong tea leaf waste on the growth of prostate cancer cells DU-145 and mice tumors remains unexplored. The objective of this study was to prepare catechin nanoemulsion from Oolong tea leaf waste and demonstrate its efficiency in inhibiting the growth of prostate cancer cells DU-145 and mice tumors induced by DU-145.

## 2. Results and Discussion

### 2.1. Extraction and HPLC Analysis of Catechins in Oolong Tea Leaf Waste

Due to the high polarity of catechins, catechins are usually extracted from tea leaves by using polar solvents such as ethanol, methanol, acetone and acetonitrile alone or in combination [6]. However, several reports have demonstrated that the extraction efficiency can be greatly improved by choosing a combination of solvents instead of a single solvent [6]. Initially, three solvent systems of 30%, 50% and 70% ethanol in water were compared for extraction efficiency of total catechins in Oolong tea leaf waste. After HPLC analysis, the highest yield of total catechins (54.22 mg/g) was shown for 50% ethanol, followed by 70% ethanol (53.3 mg/g) and 30% ethanol (24.62 mg/g). Thus, 50% ethanol was selected for extraction of catechins from Oolong tea leaf waste.

**Figure 1:**
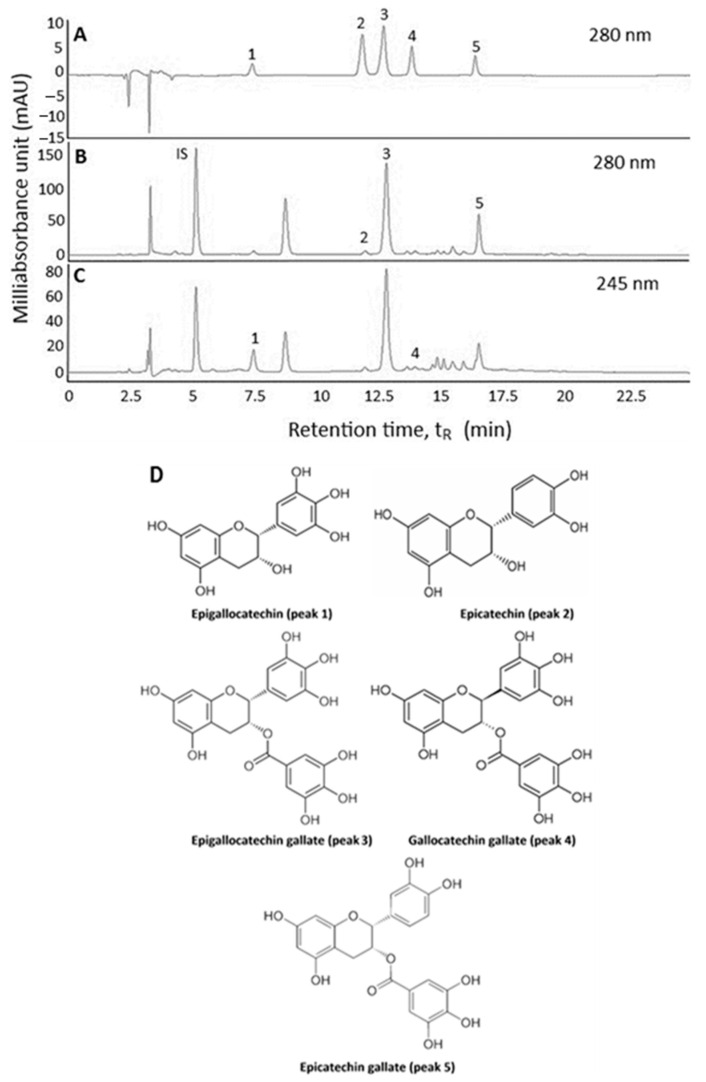
HPLC chromatograms of catechin standards (**A**) and Oolong tea leaf waste extract (**B**,**C**) at detection wavelengths 280 nm (**A**,**B**) and 245 nm (**C**), as well as the structures of 5 catechins (**D**), epigallocatechin (peak 1), epicatechin (peak 2), epigallocatechin gallate (peak 3), gallocatechin gallate (peak 4) and epicatechin gallate (peak 5). IS, internal standard (L-tryptophan).

An HPLC method based on Tsai and Chen [6] was modified to separate catechins in Oolong tea leaf waste. By using the HPLC separation conditions as described in the Methods section, a total of five catechins including EGC, EC, EGCG, GCG and ECG were separated in a mixture of standards at 280 nm (Figure 1A). However, for Oolong tea extract, three catechins including EC, EGCG and ECG were detected at 280 nm (Figure 1B), while the other two catechins EGC and GCG at 245 nm (Figure 1C) due to a lower detector response of both catechins at 280 nm. The chemical structures of all five catechins are shown in Figure 1D. Apparently, the elution order of five catechins was based on polarity, with EGC being eluted first because of highest polarity, followed by EC, EGCG, GCG and ECG. Table 1 shows the retention time (t_R_), retention factor (k), separation factor (α) and peak purity (PP) of catechins in Oolong tea leaf waste extract, which ranged from 7.4–16.42 min, 1.26–4.03, 1.10–2.08 and 99.92–99.99%, respectively. Both k and α values indicated that an adequate separation of five catechins in Oolong tea leaf waste was attained through control of an optimal solvent strength and selectivity of mobile phase towards catechin components. Furthermore, the incorporation of an acidic modifier such as formic acid into the mobile phase is necessary to overcome the problems associated with peak tailing and broadening [6]. L-tryptophan was found to be a suitable internal standard for catechin quantitation in Oolong tea leaf waste because of no interference with catechin separation, complete elution from the HPLC column, and showing similar maximum absorption wavelength of catechins. The identification data of catechins in Oolong tea leaf waste is also shown in Table 1, and a total of five catechins were identified through the comparison of maximum absorption wavelength (λ_max_) and mass spectra of unknown peaks with those of reference standards and those reported in the literature [2,6,11,12,13].

### 2.2. Method Validation

The intra-day variability for EGC, EC, EGCG, GCG and ECG was 0.39%, 1.34%, 0.2%, 0.56% and 0.91%, respectively, whereas the inter-day variability was 1.37%, 1.03%, 0.4%, 0.63% and 0.56%, respectively (Table 2). This method demonstrated high repeatability and precision. Similar outcomes were reported by Wang, Li, Wang, Li, Ling, Liu, Chen and Bi [2] and Tsai and Chen [6]. The LOD for EGC, EC, EGCG, GCG and ECG was 0.18, 0.11, 0.04, 0.03 and 0.03 μg/mL, respectively, while the LOQ was 0.55, 0.32, 0.12, 0.11 and 0.1 μg/mL (Table 2), respectively. Comparatively, this result showed a higher sensitivity than that reported by Tsai and Chen [6]. In addition, high recovery was shown for EGC, EC, EGCG, GCG and ECG, which equaled 96.6%, 94.9%, 104.2%, 86.5% and 98.1%, respectively (Table 2), revealing the high accuracy of this method. According to a method validation specification issued by the Taiwan Food and Drug Administration [14], the recovery should be in the range of 85–110% to be acceptable when the analyte concentrations are >100 ppm. Quantitation was based on the linear regression equations obtained using the standard curves, with EGCG being present in the largest amount (28.36 mg/g), followed by ECG (10.75 mg/g), EGC (7.17 mg/g), EC (6.29 mg/g) and GCG (1.67 mg/g) (Table 2). A similar outcome was reported in Oolong tea leaves by Lin, Lin, Liang, Lin-Shiau and Juan [1] and Wang, Li, Wang, Li, Ling, Liu, Chen and Bi [2].

### 2.3. Nanoemulsion Characteristics

**Figure 2:**
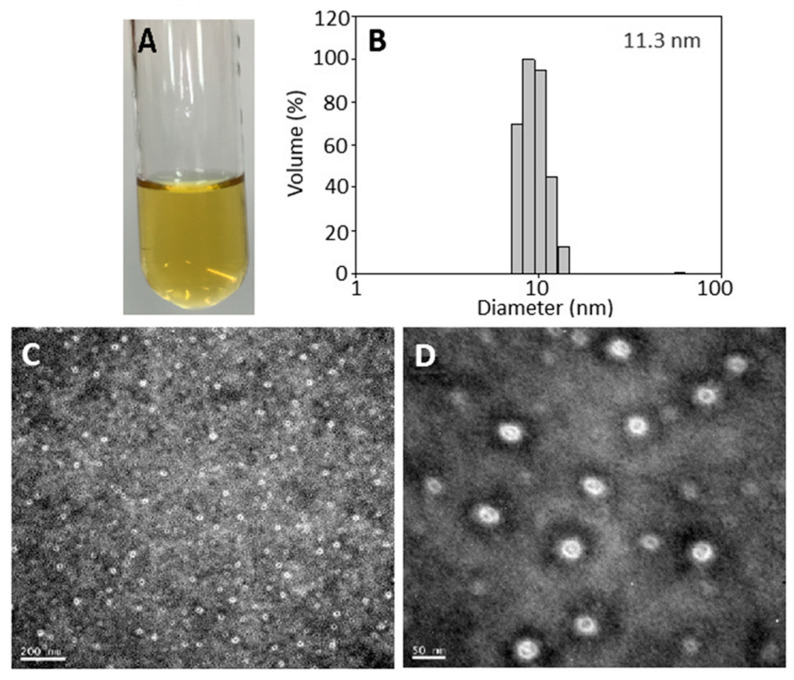
Catechin nanoemulsion containing total catechins at 1000 μg/mL with yellow appearance (**A**) along with particle size distribution as determined by dynamic light scattering method (**B**) as well as size and shape by transmission electron microscope (**C**,**D**).

A transparent catechin nanoemulsion with a yellow appearance containing total catechins at 1000 µg/mL is shown in Figure 2A. The average particle size and polydispersity index as determined by DLS was 11.3 nm and 0.229 (Figure 2B), respectively, implying a narrow particle size distribution. Accordingly, the PDI has to be controlled between 0.1–0.25 to obtain a homogeneous particle size distribution [15]. Similarly, an average particle size of 19 nm with a round shape was observed by TEM analysis (Figure 2C,D). For zeta potential, the catechin nanoemulsion was −67.2 mV, revealing that a high stability of nanoemulsion was prepared. Based on a report by Silva et al. [16], the zeta potential has to be >30 mV or <−30 mV to be stable due to static electricity repulsion between nanoparticles. More importantly, the catechin nanoemulsion with high negative zeta potential can favor interaction with a specific cationic region on cell surface for subsequent cellular uptake through endocytosis. The encapsulation efficiency of this catechin nanoemulsion was 83.4%. A similar outcome was reported by Tsai and Chen [6], who prepared a catechin nanoemulsion composed of lecithin, Tween 80 and water with green tea leaf waste as raw material, the average particle size, PDI, zeta potential and encapsulation efficiency were shown to be 11.45 nm, 0.27, −66.3 mV and 88.1%, respectively. Comparatively, the average particle size of the catechin nanoemulsion prepared in our study was much smaller than that reported by Kim et al. [17], who prepared a nanoemulsified green tea extract with an average particle size of 300 nm.

Furthermore, a minor change in particle size, PDI, zeta potential and encapsulation efficiency was shown for the catechin nanoemulsion over a 90-day storage period at 4 °C, as evident by a range of 11.2–12.2 nm, 0.229–0.287, −63.3–−67.2 mV and 81.5–83.4%, respectively. Likewise, only a minor change of particle size and zeta potential was observed for the catechin nanoemulsion when subjected to heating at 100 °C for 2 h (data not shown).

### 2.4. Effects of Solvent and Blank Nanoemulsion on DU-145 and CCD-986SK Cells

**Figure 3:**
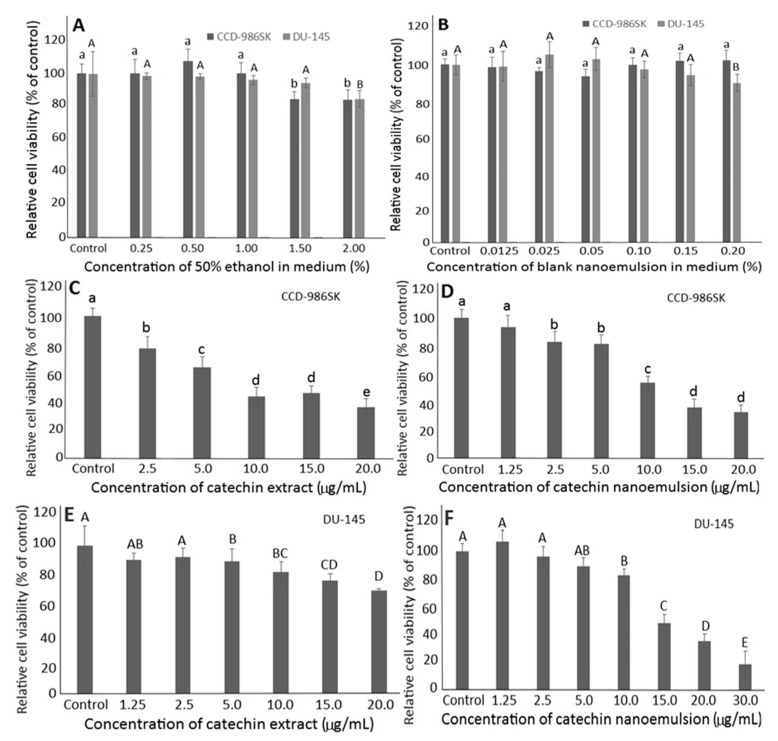
Effects of different concentrations of 50% ethanol (**A**) and catechin-free blank nanoemulsion (**B**) as well as catechin extract (**C**,**E**) and catechin nanoemulsion (**D**,**F**) on fibroblast cells CCD-986SK (**C**,**D**) and prostate cancer cells DU-145 (**E**,**F**) following incubation for 48 h. For control treatment, cells were incubated only in DMEM medium for CCD-986SK cells, and MEM medium for DU-145 cells. Data are shown as mean ± standard deviation of triplicate analyses (*n* = 3), with data bearing different small (a–e) and capital letters (A–E) to denote significantly different values at *p* < 0.05 for fibroblast cells CCD-986SK and prostate cancer cells DU-145, respectively.

Initially, the effects of the extraction solvent (50% ethanol) and the blank nanoemulsion containing lecithin, Tween 80 and water on the growth of fibroblast cells CCD-986SK and prostate cancer cells DU-145 have to be studied to avoid interference of subsequent experiments. With the dose of 50% ethanol at 0.25%–1%, the survival rates of both types of cells were ≥95.81% (Figure 3A). However, the survival rate of CCD-986SK cells followed a concentration-dependent decline after the dose of 50% ethanol was raised to be ≥1.5%. Similarly, with the concentration of blank nanoemulsion in the range of 0.0125%–0.1%, the survival rates of both DU-145 and CCD-986SK cells were ≥95.79% (Figure 3B). Following a rise in the blank nanoemulsion dose to 0.2%, the survival rate of DU-145 cells decreased to 90.18% (Figure 3B). Thus, for subsequent experiments, the doses of 50% ethanol and blank nanoemulsion were controlled at 1% and 0.1%, respectively.

### 2.5. Effects of Catechin Extract and Nanoemulsion on DU-145 and CCD-986SK Cells

Figure 3 also shows the effects of catechin extracts and nanoemulsions on the growth of CCD-986SK and DU-145 cells. A concentration-dependent decline in the survival rate of CCD-986SK cells was shown for both catechin extracts and nanoemulsions, with the IC_50_ being 9.83 and 10.23 μg/mL, respectively (Figure 3C,D). This outcome indicated that catechin extracts possessed a higher toxicity towards CCD-986SK cells than catechin nanoemulsions. Similarly, a concentration-dependent decline in survival rate of DU-145 cells was found for both catechin extracts and nanoemulsions, with the IC_50_ being 214.6 and 13.52 μg/mL, respectively (Figure 3E,F), demonstrating a more pronounced inhibition effect of catechin nanoemulsion against DU-145 cells.

In the literature reports, most studies focused on the inhibition effect of catechin standards on cancer cell growth. For instance, Ravindranath et al. [18] studied the effect of EGCG standard (11.5–45.8 μg/mL) on the growth of prostate cancer cells DU-145 and HH870, and the IC_50_ was shown to be 40.6 and 20.8 μg/mL, respectively. In a similar study, the cell survival rates of PC-3 were 73%, 63% and 46% after treatment with EGCG standard at 5.7, 11.5 and 22.9 μg/mL, respectively, with the IC_50_ being 17.9 μg/mL [19]. In another study, the IC_50_ was shown to be 26.5, 33.9 and 27.0 μg/mL for prostate cancer cells DU-145 when treated with ECG, EGCG and EGC standards, respectively [20]. In a similar study, Tsai and Chen [6] studied the effect of catechin extracts and nanoemulsions prepared from green tea leaf waste on the inhibition of prostate cancer cells PC-3 cells and the IC_50_ was shown to be 15.4 and 8.5 μg/mL, respectively. This result is similar to our finding that catechin nanoemulsion possessed a more pronounced effect in inhibiting prostate cancer cells DU-145 than catechin extract. Furthermore, compared to many published reports dealing with the effect of catechin standards on the inhibition of prostate cancer cells DU-145, a much lower IC_50_ was observed for the catechin nanoemulsion in our study, which may be accounted for by the synergistic effect of different catechins present in catechin nanoemulsion.

### 2.6. Cell Cycle Analysis

The effect of catechin extracts and nanoemulsions on cell cycle distribution of DU-145 cells is shown in Table 3. A concentration-dependent increase in sub-G1 proportion was shown and reached a plateau for both catechin extracts and nanoemulsions at 20 μg/mL. Comparatively, at the same dose, catechin nanoemulsions showed a higher sub-G1 proportion than catechin extracts, implying that the former was more effective in inducing apoptosis of DU-145 cells than the latter. The same trend was shown for the S proportion in DU-145 cells as affected by catechin nanoemulsions, revealing the possible cell cycle arrest at S phase. Interestingly, no significant difference (*p* > 0.05) in proportions of G0/G1, S and G2/M of DU-145 cells was observed when treated with catechin extracts at 10, 15 and 20 μg/mL. Likewise, for catechin nanoemulsions, the G2/M proportion showed no significant difference (*p* > 0.05) at all three doses. Although no concentration-dependent response was found for the G2/M proportion in DU-145 cells, both catechin extracts and nanoemulsions may also be effective in inhibiting DU-145 growth through the retardation of mitosis for subsequent DNA fragmentation. In a previous study, Tsai and Chen [6] also reported that the cell cycle of PC-3 cells was arrested at S and G2/M phases for both catechin extracts and nanoemulsions prepared from green tea leaf waste. Similar results were reported for the cell cycle arrest of DU-145 cells at S and G2/M phases for EGCG standard [21]. Nevertheless, in a study dealing with the effect of flavonoids on the inhibition of prostate cancer cells PC-3 and LNCaP, the cell cycle was arrested at G1 and G2/M phases [22]. Obviously, the arrest of prostate cancer cells at a certain phase can be dependent upon varieties of cell type, bioactive compound and sample extract, as well as methods of catechin nanoemulsion preparation and incubation time and dose.

### 2.7. Cell Apoptosis Analysis

Table 4 shows the proportions of necrosis cells (B1), late apoptosis cells (B2), viable cells (B3) and early apoptosis cells (B4). Compared to control, a significantly higher (*p* < 0.05) proportion of B1, B2, and B4 of DU-145 cells was shown for catechin nanoemulsions at 20 μg/mL. The same trend was observed for catechin extracts with the exception of B2, as no significant difference (*p* > 0.05) was found between control and catechin extracts at 20 μg/mL. Comparatively, catechin nanoemulsions were more effective in inhibiting the growth of DU-145 cells than catechin extracts at the same dose (20 μg/mL), as evident by a larger proportion of B1, B2 and B4 occurring during apoptosis. A similar finding was reported by Tsai and Chen [6], demonstrating that both catechin extracts and nanoemulsions prepared from green tea leaf waste could elevate proportions of B1, B2 and B4 of PC-3 cells, with the latter being more effective. Moreover, a similar outcome was observed for the effects of EGCG standard on apoptosis of prostate cancer cells DU-145 [21].

**Figure 4:**
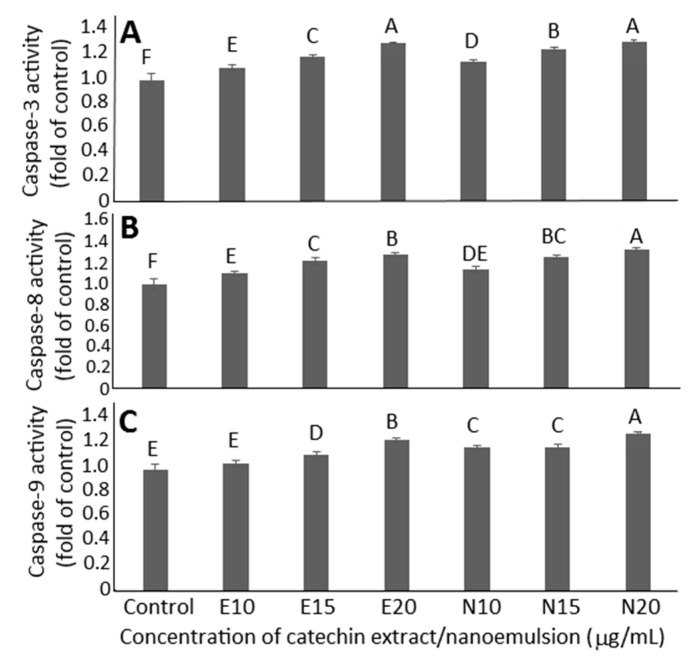
Effects of catechin extract and catechin nanoemulsion on caspase-3 (**A**), caspase-8 (**B**) and caspase-9 (**C**) activities of prostate cancer cell DU-145. For control treatment, cells were incubated only in MEM medium. E10, E15 and E20 are catechin extract treatments at 10, 15 and 20 μg/mL concentrations, respectively, while N10, N15 and N20 are catechin nanoemulsion treatments at the same concentrations. Data are shown as mean ± standard deviation of triplicate analyses (*n* = 3), with data bearing different capital letters (A–F) to denote significantly different values at *p* < 0.05.

The effect of catechin extracts and nanoemulsions on caspase-3, caspase-8 and caspase-9 activities is shown in Figure 4A–C. A concentration-dependent rise in activities of caspase-3, caspase-8 and caspase-9 was found for both catechin extracts and nanoemulsions. By comparison, at the same dose (20 μg/mL), catechin nanoemulsions showed significantly higher (*p* < 0.05) caspase-8 activity than catechin extracts (Figure 4B), but there was no significant difference in caspase-3 activity between catechin extracts and nanoemulsions at 20 μg/mL (Figure 4A). For caspase-9, a significantly higher (*p* < 0.05) activity was found for catechin nanoemulsion than for catechin extract at all three doses (Figure 4C). As caspase-3 is responsible for execution of cell apoptosis, this outcome implied that catechin nanoemulsions should be more efficient in inducing apoptosis of DU-145 cells than catechin extracts at 10 or 15 μg/mL.

Similar outcomes were reported for prostate cancer cells PC-3, DU-145 and LNCaP when treated with EGCG [22,23] or green tea polyphenol extract [24]. In another study dealing with the effect of EGCG and EGCG–chitosan nanoparticle on the growth of PC-3 and LNCaP cells, Shabana [25] reported that the activities of caspase-3, caspase-8 and caspase-9 increased for cell cycle arrest at S phase and for apoptosis to occur. Taken together, catechin nanoemulsion was more effective in inhibiting DU-145 cell proliferation than catechin extract, likely through activation of caspase-9 for initiation and then caspase-8 and caspase-3 activation for apoptosis execution of DU-145 cells through cell cycle arrest at S and G2/M phases.

### 2.8. Anti-Tumor Study of Catechin Extract, Catechin Nanoemulsion and Paclitaxel on DU-145 Induced Tumor in Mice

**Figure 5:**
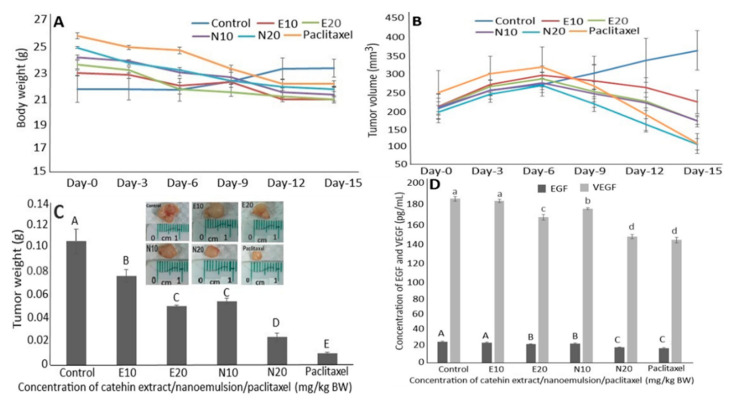
Effects of catechin extract, catechin nanoemulsion and paclitaxel on body weight (**A**), tumor volume (**B**) and tumor weight (**C**) as well as serum EGF and VEGF levels (**D**) of nude mice. E10 and E20 are catechin extract treatments at 10 and 20 mg/kg BW, respectively, with the same injection volume (0.2 mL), while N10 and N20 are catechin nanoemulsion treatments at the same dose. For paclitaxel treatment, an injection volume of 0.2 mL (10 mg/kg BW) was used. Data are shown as mean ± standard deviation of triplicate analyses (*n* = 3), with data bearing different capital letters (A–E) in Figure 5C,D and small letters (a–d) in Figure 5D to denote significantly different values at *p* < 0.05.

Based on a preliminary study, two doses (10 and 20 mg/kg BW) each of catechin extracts and catechin nanoemulsions were chosen for comparison with a single dose (10 mg/kg BW) of the positive control drug paclitaxel. Compared to the control, a slight change in mice weight was shown after five injections (injection once every 3 days) for all five treatments (Figure 5A). However, the mice tumor volume followed a time-dependent increase over a 6-day period (after two injections) for all the treatments including control (Figure 5B). After day 12, the mice tumor volume of the control treatment continued to rise while the other treatments showed a declining trend by 15.8% and 22.9% for catechin nanoemulsion at 20 mg/kg BW and paclitaxel at 10 mg/kg BW, respectively. After day 15, the mice tumor volume for the control treatment further rose by 71.1%, but declined by 17.2%, 16.7%, 41.3% and 52.5% for catechin extracts at 20 mg/kg BW, catechin nanoemulsions at 10 and 20 mg/kg BW and paclitaxel at 10 mg/kg BW, respectively. Although the nanoemulsion with high negative zeta potential provides stability, it may cause stressful pain to animals during injection. However, in our study, the injection of catechin nanoemulsion with high negative zeta potential (−67.2 mV) did not cause any noticeable discomfort or pain in mice.

Comparatively, both the high dose of catechin nanoemulsion and low dose of paclitaxel were the most effective in decreasing mice tumor volume. Similarly, compared to the control, after day 15, the mice tumor weights were reduced by 27.9%, 52.6%, 48.3%, 77.5% and 90.8% for 10 and 20 mg/kg BW of catechin extracts, 10 and 20 mg/kg BW of catechin nanoemulsions and 10 mg/kg BW of paclitaxel, respectively (Figure 5C). In addition, a dose-dependent decline in mice tumor weight was shown for all five treatments, with 20 mg/kg BW of catechin nanoemulsion and 10 mg/kg BW of paclitaxel being the most efficient. In several previous studies, Roomi et al. [26] reported that the incorporation of green tea extracts into diet could decrease tumor weight and tumor volume induced by PC-3 cells in male athymic nude mice by 47% and 53%, respectively. In a similar study dealing with the effect of intraperitoneal injection of 200 μM EGCG for 5 weeks on tumor formation in male CB17-SCID mice induced by prostate cancer cells PC-3ML, the tumor volume could be reduced by 0.6 mm [27]. Likewise, the tumor volume could be reduced by 20–30% for tumors induced by PC-3 and LNCaP 104-R cells in male athymic nude mice after intraperitoneal injection with 1 mg EGCG for 2 weeks, with EGCG being more effective in inhibiting LNCaP 104-R cells (androgen dependent) than PC-3 cells (non-androgen dependent) [28].

### 2.9. Regulation of EGF and VEGF Levels in Serum

Both epidermal growth factor (EGF) and vascular endothelial growth factor (VEGF) are vital parameters in regulating cell growth and necrosis. The former can conjugate with EGF receptor for regulation of protein expression while the latter can promote angiogenesis for tumor growth. Figure 5D shows the effects of catechin extracts, catechin nanoemulsion and paclitaxel on serum EGF and VEGF levels in nude mice. Compared to control, a dose-dependent decline was shown in EGF and VEGF levels for both catechin extract and nanoemulsion treatments. Comparatively, both catechin nanoemulsion (20 mg/kg BW) and paclitaxel (10 mg/kg BW) were the most effective in decreasing EGF and VEGF levels. This result is in agreement with that shown above for the reduction in both tumor weight and volume.

It may be postulated that the small size of catechin nanoemulsion may be more readily penetrated into cancer cells through EPR effect by passive targeting for accumulation in tumor tissues [8]. In several previous studies, Roomi, Ivanov, Kalinovsky, Niedzwiecki and Rath [26] also observed a reduction in serum VEGF level in tumors of male athymic nude mice induced by PC-3 cells after the incorporation of green tea extract into diet and feeding for 4 weeks. In our study both treatments of catechin nanoemulsion at 20 μg/mL and paclitaxel at 10 μg/mL possessed a pronounced inhibition effect on the growth of mice tumors through a decline in both serum EGF and VEGF levels, leading to retardation of angiogenesis in tumors. This finding demonstrated a comparable anti-tumor effect of catechin nanoemulsion to that of paclitaxel.

## 3. Materials and Methods

### 3.1. Materials

A total of 12 kg of Oolong tea leaf waste (*Camellia sinensis* (L.) kuntze var.) were provided by a tea beverage processing company in Tainan, Taiwan. Prior to use, the Oolong tea leaf waste was freeze dried and then poured into 8 bags and vacuum sealed separately and stored in a −30 °C freezer. A total of 8 catechin standards including catechin, epicatechin, gallocatechin, epigallocatechin, gallocatechin gallate, epigallocatechin gallate, catechin gallate and epicatechin gallate, as well as internal standard L-tryptophan, were procured from Sigma-Aldrich Co. (St. Louis, MO, USA). The HPLC grade solvents methanol and acetonitrile were obtained from Merck Co. (Darmstadt, Germany). Both ethanol (99.9%) and formic acid as well as potassium dihydrogen phosphate were obtained from Sigma-Aldrich Co. Deionized water was madeusing a Milli-Q water purification system from Millipore Co. (Bedford, MA, USA). Lecithin was obtained from Cheng-Fung Co. (Taipei, Taiwan) while Tween 80 was acquired from Yi-Pa Co. (Taipei, Taiwan). The fixation solution (2.5% glutaraldehyde, 4% formaldehyde and 1% osmium tetroxide) and buffer solution (0.1 M Soreneon’s phosphate buffer) for TEM sample preparation were procured from Sigma-Aldrich Co., while the dehydration reagents (30%, 50%, 70%, 95% and 100% ethanol, and acetone), Spurr’s resin kit and EMS G300-Cu copper grid were obtained from Electron Microscopy Sciences (Hatfield, PA, USA).

Human prostate cancer cells (DU-145) and human fibroblast cells (CCD-986SK) with research resource identifier (RRID) of CVCL_0105 and CVCL_2400, respectively, were purchased from Bioresources Collection and Research Center, Taiwan Food Industry Development and Research Institute (Hsinchu, Taiwan). All experiments were performed with mycoplasma-free cells. The reagents for cell culture including sodium pyruvate, non-essential amino acid and penicillin-streptomycin were acquired from Gibco Co. (CA, USA), while minimum essential medium (MEM), Dulbecco’s modified eagle’s medium (DMEM), fetal bovine serum (FBS) and 0.25% trypsin-EDTA were from HyClone Co. (Longan, UT). Disodium hydrogen phosphate was procured from Panreac Quimica Co. (Needham Market, Suffolk, UK). Dimethyl sulfoxide (DMSO), hydrochloric acid, propidium iodide (PI), were obtained from Sigma-Aldrich Co. The MTT (3-(4,5)-dimethylthiazol-2-yl)-2,5-diphenyltetrazolium bromide) reagent was obtained from USB Co. (Cleveland, OH, USA). Both caspase-3 assay kit and FITC Annexin V apoptosis detection kit were from BD Bioscience Co. (San Jose, CA, USA), while caspase-8 and caspase-9 fluorometric assay kits were from Bio Vision Co. (Milpitas, CA, USA).

For the animal study, a total of 36 4-week-old male BALB/c nude mice (specific pathogen free) were obtained from the National Experimental Animal Center (Taipei, Taiwan). Mouse EGF ELISA kit and VEGF ELISA kit were from Koma Biotech Co. (Seoul, Korea), while paclitaxel was obtained from Sigma-Aldrich Co.

### 3.2. Extraction of Catechins from Oolong Tea Leaf Waste

A method based on Tsai and Chen [6] was modified to extract catechins from Oolong tea leaf waste. Briefly, a 0.2 g sample of Oolong tea leaf waste powder was mixed with 4 mL of 30%, 50% or 70% ethanol in water for comparison of extraction efficiency. Then, the mixture was sonicated in an ultrasonicator (DC400H, Hua-Hsia Co., Taipei, Taiwan) at room temperature for 1 h, followed by centrifuging in a high-speed centrifuge (Sorvall RC6 plus, Thermo Fisher Scientific Co., San Jose, CA, USA) at 4000 rpm (25 °C) for 30 min and collecting the supernatant, which was then filtered through a 0.6 μm glass filter paper, evaporated to dryness under vacuum, dissolved in 5 mL of 50% ethanol in water, filtered through a 0.22 μm membrane filter and stored at −20 °C for use.

### 3.3. HPLC Separation of Catechins

The separation condition of catechins in Oolong tea leaf waste was based on a report by Tsai and Chen [6] and modified. An HPLC system from Agilent (model 1200 series, Agilent Technologies Co., Santa Clara, CA, USA) coupled with a single quadrupole mass spectrometer (model 6130) and a Gemini C18 column (250 × 4.6 mm ID, 5 μm particle size) from Phenomenex Co. (Torrance, CA, USA) was used with flow rate at 1 mL/min, column temperature at 30 °C, detection wavelengths at 245 nm and 280 nm and a gradient solvent system of 0.1% formic acid solution (A) and acetonitrile (B) was used: 88% A and 12% B initially, maintained for 3 min, then changed to 80% A and 20% B in 9 min, 75% A and 25% B in 12 min, maintained for 13 min, and returned to the original ratio. A total of five catechins in Oolong tea leaf waste were separated within 17 min.

### 3.4. Identification of Catechins

For identification of the various catechins in Oolong tea leaf waste, both retention time and mass spectra of unknown peaks in the HPLC chromatogram were compared with authentic standards and those reported in the literature. The peak purity was automatically determined by a photodiode array detector. A single quadrupole mass spectrometer with ESI mode (negative) was used for identification with the same parameters as reported by Tsai and Chen [6]. For positive identification, an HPLC-MS/MS instrument with ESI mode (LTQ Orbitrap XL, Thermo Fisher Scientific Co., San Jose, CA, USA) was used in the scanning range from 100–1000 m/z with heated temperature at 300 °C, sheath gas flow rate at 50 arbitrary units, aux gas flow rate at 25 arbitrary units, sweep gas flow rate at 0 arbitrary units, spray voltage at 3 kV, capillary temperature at 275 °C, capillary voltage at −35 V and tube lens voltage at −100 V.

### 3.5. Validation of Quality Control Parameters

Both intra-day and inter-day variabilities were determined for method validation [29], with the former being conducted by collecting catechin sample solution containing the internal standard L-tryptophan (10 μg/mL) for analysis in the morning, afternoon and evening on the same day in triplicate for a total of 9 analyses, while the latter was carried out by collecting catechin sample solution containing L-tryptophan (10 μg/mL) for analysis in the morning, afternoon and evening on the first, second and third day in triplicate, separately, for a total of 27 analyses.

For LOD and LOQ determination, three concentrations (0.2, 0.4 and 0.6 μg/mL) in 50% ethanol were prepared for four catechin standards including EGC, EC, EGCG and GCG separately. For ECG, 0.2, 0.25 and 0.3 μg/mL were prepared. Each concentration was injected into HPLC three times and the standard curves were prepared by plotting concentration against average peak area; from the slope (S) and maximum noise height (N), both LOD and LOQ were calculated based on S/N ≥ 3 and S/N ≥ 10, respectively [6].

The recovery was determined by adding two levels of catechin standards including EGC (700 and 1050 μg), EC (600 and 900 μg), EGCG (3000 and 4500 μg), GCG (150 and 225 μg) and ECG (1000 and 1500 μg) to 0.2 g of Oolong tea leaf waste samples for extraction and HPLC analysis. The recovery was obtained based on the relative ratio of catechin level after HPLC to that before HPLC.

### 3.6. Quantitation of Catechins

For quantitation, a total of seven concentrations were prepared for EGC (85, 100, 115, 130, 145, 160 and 175 μg/mL), EC (40, 45, 50, 55, 60, 65 and 70 μg/mL), EGCG (355, 370, 385, 400, 415, 430 and 445 μg/mL), GCG (5, 10, 15, 20, 25, 30 and 35 μg/mL) and ECG (85, 100, 115, 130, 145, 160 and 175 μg/mL). Then, each concentration was mixed with L-tryptophan at a fixed concentration at 10 μg/mL. After injection into HPLC three times for each concentration, the standard calibration curves were prepared by plotting the concentration ratio (standard versus IS) against the area ratio (standard versus IS), and both the linear regression equations and coefficient of determination (R^2^) were automatically obtained by a Microsoft EXCEL software system. The contents of each catechin (μg/g) were then quantified [6].

### 3.7. Preparation of Catechin Nanoemulsion

A sample of catechin extract (0.892 mL) containing catechin at 11,208.2 μg/mL was poured into a tube and evaporated to dryness under nitrogen. Then, 0.05 g of lecithin in liquid form (0.5%) and 0.7 g of Tween 80 (7%) were added sequentially with thorough stirring after each addition, followed by adding 9.25 g deionized water (92.5%), stirring thoroughly and sonicating the solution for 1.5 h to obtain a transparent catechin nanoemulsion of 10 mL with a yellow appearance containing total catechins at 1000 μg/mL.

### 3.8. Characterization of Catechin Nanoemulsion

A portion of catechin nanoemulsion (100 μL) was collected and diluted 30 times with 25 mM of potassium dihydrogen phosphate buffer solution (pH 5.3–5.5), after which the solution was poured into a polystyrene tube and the particle size distribution and mean particle size were determined by a dynamic light scattering instrument (DLS) from Brookhaven Instruments Co. (Holtsville, NY, USA) with a BIC Particle Sizing 90 Plus software system. A portion of catechin nanoemulsion (100 μL) was diluted with deionized water 30 times, after which 300 μL were collected and the zeta potential was determined in a SZ-100 model zeta potential analyzer from Horiba Scientific Co. (Kyoto, Japan). A portion of catechin nanoemulsion was diluted with deionized water 100 times, after which 20 μL were collected and dropped onto a copper grid for 45 s and the excessive sample was removed with a glass filter paper. Then, 2% (20 μL) phosphotungstic acid (PTA) was added for negative staining for 30 s, and the excessive PTA was removed with a glass filter paper and sample placed in a moisture-proof box for drying. Both the size and shape of catechin nanoemulsions were determined by a JEM-1400 model TEM (JEOL Co., Tokyo, Japan) by enlarging the sample solution 3 × 10^5^ times and observing it under 120 kV. In addition, a portion (100 μL) of catechin nanoemulsion was collected and diluted 10 times with 25 mM potassium dihydrogen phosphate buffer solution (pH 5.3–5.5). Then, the solution was poured into a centrifuge tube containing 3 kDa of dialysis membrane for centrifugation in a DSC-301SD LED model microcentrifuge (Digisystem Laboratory Instruments Co., New Taipei City, Taiwan) at 12,000 rpm (25 °C) for 20 min. Unencapsulated (free) catechin could penetrate into the dialysis membrane, and the lower layer (200 μL) was collected and evaporated to dryness under nitrogen, and 100 μL internal standard (10 μg/mL) was added for HPLC analysis. The encapsulation efficiency was calculated using the following formula:

(1) $$\mathrm{Encapsulation}\;\mathrm{efficiency}\;\left(\%\right)=\frac{{\left(\mathrm{catechin}\right)}_{\mathrm{total}}-{\left(\mathrm{catechin}\right)}_{\mathrm{free}}}{{\left(\mathrm{catechin}\right)}_{\mathrm{total}}}\times 100$$

For the stability study, catechin nanoemulsion was stored at 4 °C for 90 days, during which a portion of the sample was collected every 15 days for the determination of particle size distribution by DLS and zeta potential. Also, a portion of catechin nanoemulsion (200 μL) was poured into a tube and placed into a water bath with temperatures controlled at 40, 50, 60, 70, 80, 90 and 100 °C separately and heated for 0.5, 1, 1.5 and 2 h. Both particle size distribution and zeta potential were also analyzed.

### 3.9. Cell Culture for Prostate Cancer Cells DU-145

Prostate cancer cells DU-145 were cultured in MEM medium by mixing 10.1 g MEM powder with 700 mL deionized water, 2.2 g sodium bicarbonate and 80 mL FBS, 10 mL penicillin-streptomycin, and diluted to 1 L with deionized water (pH 7.2–7.4). Similarly, human fibroblast cells CCD-986SK were cultured in DMEM medium, prepared by mixing 700 mL deionized water with 1 L medium powder, 1.5 g sodium bicarbonate, 100 mL FBS, 10 mL penicillin-streptomycin and 10 mL sodium pyruvate (100 mM), and diluted to 1 L with deionized water (pH 7.2–7.4). Both media were filtered through a 0.2 μm membrane filter and stored at 4 °C for use. For subculture of DU-145 or CCD-986SK cells, the medium in a culture plate was removed, washed with PBS and one mL of 0.25% trypsin-EDTA was added for incubation (SCA-165DS CO_2_ incubator, Astec Co., Fukuoka, Japan) for 3–5 min. Then, 1 mL of medium was added to terminate trypsin-EDTA reaction, after which the medium was centrifuged at 1500 rpm (25 °C) for 5 min and the supernatant was aspirated. Then, 1 mL of MEM or DMEM was added, followed by collecting an appropriate amount of cells for seeding in a culture plate containing fresh medium.

### 3.10. MTT Assay for DU-145 and CCD-986SK Cells

A 50 mg MTT powder was dissolved in 10 mL of sterilized PBS and filtered through a 0.22 μm membrane filter to obtain a stock solution of 5 mg/mL. Prior to use, the MTT stock solution was mixed with Hank’s balanced salt solution (HBSS) at 1:9 (*v*/*v*). Cells were seeded in a 96-well plate with each well containing 1 × 10^4^ cells. After incubation overnight for cell attachment, the medium was removed and replaced with various concentrations of catechin extract or catechin nanoemulsion for 48 h incubation. Then, the medium was discarded and 200 μL of MTT (0.5 mg/mL in PBS) were added and cultured for 2 h, after which the MTT solution was removed, 100 μL of DMSO was added and stirred for 10 min and the absorbance was measured at 570 nm with an ELISA reader (Molecular Devices Co., Sunnyvale, CA, USA). The relative cell survival rate was calculated using a formula as described by Huang, Wei, Inbaraj and Chen [8].

### 3.11. Cell Cycle Analysis for DU-145 Cells

Cells were seeded in a 6-well plate with each well containing 1 × 10^6^ cells. After incubation overnight for cell attachment, the medium was removed and replaced with various concentrations of catechin extract or nanoemulsion for 48 h incubation. Then, the medium was transferred to a centrifuge tube, washed with PBS and 0.5 mL of trypsin-EDTA (0.25%) was added for a 10 min reaction. After centrifugation at 1500 rpm for 5 min (4 °C), the supernatant was removed and the medium washed with PBS (0.5 mL) twice. Next, one mL of 70% ethanol (4 °C) was added for cell fixation at 4 °C, centrifuged at 1500 rpm for 5 min (4 °C) and the supernatant was removed, followed by washing with 0.5 mL of PBS twice, adding 0.8 mL of PBS, 0.1 mL of RNase (1 mg/mL) and 0.1 mL of propidium iodide (100 μg/mL) for reaction in a water bath for 30 min in the dark. After filtration through a 40 μm nylon filter, cells were analyzed for the proportions of sub-G1, G0/G1, S and G2/M phases by a flow cytometer (Beckman Coulter Co., Tokyo, Japan) with a Partec Flow Maz version 2.4d software system.

### 3.12. Annexin V/propidium Iodide Staining Assay for DU-145 Cells

Cells were seeded in a 6-well plate with each well containing 1 × 10^6^ cells. After incubation overnight for cell attachment, the medium was removed and replaced with various concentrations of catechin extract or nanoemulsion for 48 h incubation. Then, the medium was transferred to a centrifuge tube, washed with PBS, and 0.5 mL of trypsin-EDTA (0.25%) added for 10 min reaction. After centrifugation at 1500 rpm for 5 min (4 °C), the supernatant was removed and the medium washed with PBS twice. Then, 0.1 mL of binding buffer was added to suspend cells, followed by adding 5 μL of Annexin V-FITC and 10 μL of PI for 15 min reaction in the dark and subsequent analysis of apoptotic and necrotic cell populations by a flow cytometer with a Partec Flow Maz version 2.4d software system.

### 3.13. Activities of Caspase-3, Caspase-8 and Caspase-9

The activity of caspase-3 was determined by a fluorescence assay kit. Briefly, a sample of DU-145 cell lysate (25 μL) containing 40 μg cell protein was mixed with 100 μL of 1XHEPES buffer, after which the solution was reacted in a 37 °C water bath for 1 h in the dark and then transferred to a 96-well plate for absorbance measurement at 380 nm (excitation wavelength) and 440 nm (emission wavelength) by using a multimode microplate reader. Similarly, both caspase-8 and caspase-9 activities were determined by collecting 50 μL of DU-145 cell lysate separately and mixing with 50 μL of 2X reaction buffer, reacted in a 37 °C water bath for 1 h in the dark and then transferred to a 96-well plate for absorbance measurement at 400 nm (excitation wavelength) and 505 nm (emission wavelength) by using a multimode microplate reader (Infinite 200PRO, Tecan Co., Mannedorf, Switzerland).

### 3.14. Animal Study Approval and Handling for Evaluating Anti-Tumor Efficiency in Mice

A total of 36 4-week-old SPF (specific pathogen free) male BALB/c nude mice were obtained from the National Animal Research Center (Taipei, Taiwan) and transported to Fu Jen University Animal Research Center. A prior approval of animal experiment protocols was obtained from Fu Jen Catholic University Animal Subjects Review Committee and strict regulations on human care for laboratory animals was adopted based on approved guidelines [30]. Each mouse was raised in an individual ventilated cage and fed with LabDiet 5010 with temperature at 21 ± 2 °C and relative humidity at 55 ± 10% for 12 h under light and 12 h in the dark. All the procedures followed the standard operation method for experimental animals.

### 3.15. Animal Experiments

After an adaptation period of 2 weeks, about 1 × 10^7^ prostate cancer cells DU-145 were injected into flank of each mice for 28 days to induce tumor formation, then the tumor size was measured with a caliper every 3 days. After the average tumor volume reached about 200 mm^3^, we started the injection of catechin extract and catechin nanoemulsion. Both tumor size and mice weight were recorded every 3 days. Tumor volume was calculated using the following formula:

(2) $$V=\frac{L\times W^{2}}{2}$$

where V, L and W denote tumor volume, length and width, respectively. Then, 36 male BALB/c nude mice were divided into 6 groups with 6 mice each and with intraperitoneal injection (IP) every 3 days for a total of 6 injections: (1) control group—injection with 0.2 mL of saline solution (0.85%), (2) drug group—injection with 0.2 mL of paclitaxel in deionized water (10 mg/kg BW), (3) low dose (10 mg/kg BW) of catechin extract group—injection with 0.2 mL of catechin extract (evaporated to dryness and dissolved in deionized water), (4) high dose (20 mg/kg BW) of catechin extract group—injection with 0.2 mL of catechin extract (evaporated to dryness and dissolved in deionized water), (5) low dose (10 mg/kg BW) of catechin nanoemulsion group—injection with 0.2 mL of catechin nanoemulsion and (6) high dose (20 mg/kg BW) of catechin nanoemulsion group—injection with 0.2 mL of catechin nanoemulsion. Then, all the mice were placed under euthanasia with CO_2_ and the tumors were collected for weight measurement. The heart blood was collected and poured into a tube for centrifugation at 3500 rpm (4 °C) for 20 min, and serum in the upper layer was collected in a tube for storage at −80 °C.

### 3.16. Determination of Growth Factors in Serum

Both levels of epidermal growth factor (EGF) and vascular endothelial growth factor (VEGF) in serum were measured with an ELISA kit. In brief, a 96-well plate containing antibody was washed with 300 μL of wash buffer (0.05% Tween 20 in PBS) 4 times, and a volume (100 μL) of EGF or VEGF standard with various concentrations (15.625, 31.25, 62.5, 125, 250, 500 and 1000 pg/mL) or serum was added to the 96-well plate for a 2 h reaction at room temperature. Then, the plate was washed with 300 μL of wash buffer 4 times, followed by adding 100 μL of detection antibody for a 2 h reaction and then 100 μL of streptavidin-HRP conjugate for a 30 min reaction. The plate was washed again with 300 μL of wash buffer 4 times, after which 100 μL of the solution containing tetramethyl benzidine (TMB) was added for 5 min reaction and then 100 μL of stop buffer (2M H_2_SO_4_) was added to terminate the reaction. The absorbance was measured at 450 nm and both levels of EGF and VEGF were obtained based on the linear regression equations of the standard curves.

### 3.17. Statistical Analysis

All the data were analyzed using the statistical analysis system and subjected to ANOVA analysis and Duncan’s multiple range test for significance in mean comparison (*p* < 0.05).

## 4. Conclusions

In conclusion, an HPLC gradient mobile phase of 0.1% formic acid aqueous solution (A) and acetonitrile (B) was developed to separate five catechins within 17 min, which were identified as EGC, GCG, EC, EGCG and ECG. A highly stable catechin nanoemulsion was prepared using an appropriate proportion of lecithin, Tween 80 and deionized water. Both the catechin extracts and nanoemulsions were effective in inhibiting the growth of prostate cancer cells DU-145 through activation of caspase-9, caspase-8 and caspase-3, with the cell cycle arrest at S and G2/M phases. A larger proportion of necrosis cells, late apoptosis cells and early apoptosis cells was shown for catechin nanoemulsions than for catechin extracts during apoptosis. Both catechin nanoemulsion at 20 mg/kg BW and paclitaxel at 10 mg/kg BW were the most effective in reducing tumor volume and weight in mice through a decline in both EGF and VEGF levels in serum. Collectively, the catechin nanoemulsion prepared in this study may be used as a basis for future clinical trials for the treatment of patients with prostate cancer.

## Figures and Tables

**Table 1:** Retention time (t_R_), retention factor (k), separation factor (α), peak purity (PP), absorption wavelength and mass spectra data of catechins in Oolong tea leaf waste extract.

Peak No.	Compound	t_R_	k	α	PP (%)	Absorption Wavelength (λ_max_, nm)			Mass Spectra ([M − H]^−^, m/z) ^d^			Tandem Mass Fragment Ions (MS^2^)	
						Extract ^c^	Standard	Reported	Extract	Standard	Reported	Extract	Reported
IS ^a^	L-tryptophan	5.11	-	-	-	-	-	-	-	-	-	-	-
1	epigallocatechin (EGC)	7.40	1.26	2.08 (1,2) ^b^	99.95	270	270	271 ^e^	305	305	305 ^e^	261, 219, 137	261, 219 ^h^, 137 ^g^
2	epicatechin (EC)	11.88	2.63	2.08 (1,2) ^b^	99.94	278	278	275 ^f^	289	289	289 ^f^	245, 203, 125	245, 203, 125 ^i^
3	epigallocatechin gallate (EGCG)	12.72	2.89	1.10 (2,3) ^b^	99.98	274	274	274 ^e^	457	457	457 ^e^	331, 305, 169, 125	331, 305, 169 ^i^, 125 ^g^
4	gallocatechin gallate (GCG)	13.86	3.24	1.12 (3,4) ^b^	99.92	274	274	274 ^e^	457	457	457 ^e^	305, 169, 125	305, 169, 125 ^g^
5	epicatechin gallate (ECG)	16.42	4.03	1.24 (4,5) ^b^	99.99	276	278	278 ^e^	441	441	441 ^e^	289, 169	289, 169 ^h^

^a^ IS = internal standard. ^b^ Numbers in parentheses represent values between two neighboring peaks. ^c^ A gradient mobile phase of acetonitrile and 0.1% formic acid in water was used. ^d^ Determined by LC-MS. ^e^ Based on a reference by Tsai and Chen [6]. ^f^ Based on a reference by Wu, Xu, Héritier and Andlauer [11]. ^g^ Based on a reference by Wang, Li, Wang, Li, Ling, Liu, Chen and Bi [2]. ^h^ Based on a reference by Rio, Stewart, Mullen, Burns, Lean, Brighenti and Crozier [12]. ^I^ Based on a reference by Bastos, Saldanha, Catharino, Sawaya, Cunha, Carvalho and Eberlin [13].

**Table 2:** Quality control data of catechins in Oolong tea leaf waste by HPLC.

Compound	Calibration Curve	LOD ^b^ (μg/mL)	LOQ ^c^ (μg/mL)	Content (mg/g) ^d^	Recovery Data (%)					Intra-Day Variability		Inter-Day Variability	
					Original (μg)	Spiked (μg)	Found (μg)	Mean Recovery (%) ^e^	RSD ^f^ (%)	Content (mg/g) ^g^	RSD ^f^ (%)	Content (mg/g) ^h^	RSD ^f^ (%)
epigallocatechin (EGC)	y = 0.2551x + 0.2682 (0.9971) ^a^	0.18	0.55	7.17 ± 0.10	769.6 769.6	700.0 1050.0	1414.1 1830.9	96.6 ± 4.2	4.31	7.28 ± 0.03	0.39	7.17 ± 0.10	1.37
epicatechin (EC)	y = 0.1572x + 0.1895 (0.9966) ^a^	0.11	0.32	6.29 ± 0.06	897.4 897.4	600.0 900.0	1453.1 1772.0	94.9 ± 8.2	8.63	6.33 ± 0.09	1.34	6.29 ± 0.07	1.03
epigallocatechin gallate (EGCG)	y = 1.7592x − 28.732 (0.9909) ^a^	0.04	0.12	28.36 ± 0.08	4524.9 4524.9	3000.0 4500.0	7616.7 9261.6	104.2 ± 2.2	2.09	29.25 ± 0.06	0.20	29.15 ± 0.12	0.40
gallocatechin gallate (GCG)	y = 0.7163x − 0.0299 (0.9982) ^a^	0.03	0.11	1.67 ± 0.01	175.6 175.6	150.0 225.0	312.7 358.9	86.5 ± 2.4	2.75	1.67 ± 0.01	0.56	1.67 ± 0.01	0.63
epicatechin gallate (ECG)	y = 1.1243x − 3.0065 (0.9922) ^a^	0.03	0.10	10.75 ± 0.04	1361.6 1361.6	1000.0 1500.0	2356.4 2812.4	98.1 ± 0.2	0.15	13.11 ± 0.12	0.91	13.14 ± 0.07	0.56

^a^ Coefficient of determination (R^2^). ^b^ Limit of detection (LOD). ^c^ Limit of quantitation (LOQ). ^d^ Data are shown as mean of triplicate analyses ± standard deviation. ^e^ Mean recovery ± standard deviation of two catechin concentrations spiked and recovery determined using the formula: recovery (%) = ((amount of standard found—original amount in sample)/amount of standard spiked) × 100. ^f^ Relative standard deviation (RSD, %) = (standard deviation/mean) × 100. ^g^ Mean of 9 analyses ± standard deviation performed at three each in the morning, afternoon and evening of the same day. ^h^ Mean of 27 analyses ± standard deviation performed at three each in the morning, afternoon and evening for three consecutive days.

**Table 3:** Effect of different concentrations of catechin extract and catechin nanoemulsion on cell cycle distribution of prostate cancer cell DU-145 ^a^.

Concentration (μg/mL)	Sub-G1 (%)	G0/G1 (%)	S (%)	G2/M (%)
Control	0.98 ± 0.2 ^a^	88.82 ± 1.4 ^a^	3.63 ± 0.6 ^a^	6.43 ± 0.5 ^a^
Extract				
10	0.98 ± 0.1 ^a^	81.36 ± 1.1 ^b^	8.66 ± 1.1 ^b^	8.75 ± 0.9 ^b^
15	1.42 ± 0.1 ^b^	83.11 ± 1.6 ^b^	6.92 ± 0.8 ^b^	8.35 ± 0.6 ^b^
20	1.56 ± 0.2 ^bc^	82.73 ± 0.8 ^b^	7.96 ± 1.0 ^b^	8.19 ± 0.7 ^b^
Nanoemulsion				
10	1.47 ± 0.1 ^b^	81.95 ± 1.0 ^b^	8.22 ± 0.7 ^b^	8.14 ± 0.8 ^b^
15	1.74 ± 0.1 ^cd^	79.63 ± 0.9 ^c^	10.42 ± 0.3 ^c^	7.42 ± 0.4 ^b^
20	1.91 ± 0.1 ^d^	77.59 ± 1.2 ^c^	12.86 ± 0.4 ^d^	7.60 ± 0.4 ^b^

^a^ Data are shown as mean ± standard deviation (*n* = 3), with data bearing different small letters (a–d) in the same column denoting significantly different values at *p* < 0.05.

**Table 4:** Apoptosis of prostate cancer cells DU-145 treated with catechin extract and catechin nanoemulsion at 20 μg/mL ^a^.

Treatment	Necrosis Cells (B1) (%)	Late Apoptosis Cells (B2) (%)	Viable Cells (B3) (%)	Early Apoptosis Cells (B4) (%)
Control	0.44 ± 0.1 ^a^	1.01 ± 0.1 ^a^	97.69 ± 1.2 ^a^	0.85 ± 0.2 ^a^
Extract (20 μg/mL)	1.01 ± 0.1 ^b^	1.05 ± 0.2 ^a^	93.30 ± 0.9 ^b^	4.64 ± 0.4 ^b^
Nanoemulsion (20 μg/mL)	2.45 ± 0.2 ^c^	3.51 ± 0.6 ^b^	89.28 ± 0.7 ^c^	4.76 ± 0.1 ^b^

^a^ Data are shown as mean ± standard deviation (*n* = 3), with data bearing different small letters (a–c) in the same column denoting significantly different values at *p* < 0.05.

## Data Availability

The data presented in this study are available in this article.
